# Self-assembled non-steroidal anti-inflammatory drug-peptide hydrogel to effectively mitigate ocular inflammation

**DOI:** 10.1080/10717544.2026.2677302

**Published:** 2026-05-26

**Authors:** Yuhan Hu, Yu Li, Yuqin Wu, Yutuo Zhu, Jinrun Chen, Yinghao Ding, Zhimou Yang, Xingyi Li

**Affiliations:** a Key Laboratory of Bioactive Materials, Ministry of Education, College of Life Sciences, State Key Laboratory of Medicinal Chemical Biology, Collaborative Innovation Center of Chemical Science and Engineering, and National Institute of Functional Materials, Nankai University, Tianjin, China; b National Engineering Research Center of Ophthalmology and Optometry, Zhejiang Key Laboratory of Ophthalmic Drug Discovery and Medical Device Research, Eye Hospital, Wenzhou Medical University, Wenzhou, China

**Keywords:** Uveitis, drug-peptide conjugate, supramolecular hydrogel, ocular drug delivery, topical instillation

## Abstract

Pranoprofen (Pra) eye drop has been widely used in clinical practice for the efficient mitigation of ocular inflammation, but it often suffers from poor bioavailability owing to the existence of various ocular barriers (i.e. tear barrier and corneal epithelial barrier). In this study, we rationally designed and synthesized a Pra-peptide conjugate using a D-form tripeptide (i.e. phenylalanine-phenylalanine-aspartic acid; ffd) motif, and the resulting conjugate (i.e. Pra-ffd) spontaneously self-assembled into single-component supramolecular hydrogel for efficient mitigation of ocular inflammation. The generated hydrogel was thoroughly characterized using a rheometer and transmission electron microscopy (TEM). The proposed Pra-ffd hardly caused cytotoxicity in RAW 264.7, L929, and human corneal epithelial cells (HCEC). The Pra-ffd hydrogel dose-dependently reduced the levels of proinflammatory cytokines (e.g. PGE_2_, TNF-*α*, and IL-6) and intracellular reactive oxygen species (ROS) accumulation in lipopolysaccharide (LPS)-activated RAW 264.7 macrophage. In addition, topical instillation of the Pra-ffd hydrogel exhibited good ocular tolerance and prolonged precorneal retention (>20 min), resulting in higher bioavailability compared with the Pra solution. Using an endotoxin-induced uveitis (EIU) rabbit model, we demonstrated that topical instillation of Pra-ffd hydrogel effectively mitigated the inflammatory response in the anterior chamber and significantly reduced the entry of inflammatory cells from the circulation by preserving the integrity of the blood-aqueous humor barrier. Overall, the Pra-ffd hydrogel represents a promising ocular drug delivery platform for the treatment of inflammatory ocular disorders.

## 1. Introduction

Uveitis, a category of ocular disorders, accounts for 5%–10% of all visual impairments worldwide (Selmi 2014; E.C. and Zierhut 2021; Maghsoudlou et al. 2025). Uveitis can generally be divided into infectious and noninfectious subtypes. The management of non-infectious uveitis is more complex than that of infectious uveitis (Fan et al. 2023). Suppression of the local immune response by different therapeutic agents is a clinical mainstay for non-infectious uveitis therapy (Airody et al. 2016; Valdes and Sobrin 2020; Balasubramaniam et al. 2022; Zhang and Zhang 2023). Topical administration of glucocorticoids via instillation, intravitreal injection, and sub-conjunctival and periocular routes is still the first-line management choice for non-infectious uveitis, owing to its robust anti-inflammatory and immunosuppressive activity (Luo et al. 2019; Clarke et al. 2024; Belletti et al. 2025). However, the high risk of side effects, including intraocular pressure (IOP) elevation and cataract formation, associated with glucocorticoid therapy has limited its long-term application (Friedman et al. 2013; Yalcinsoy et al. 2022; Jian et al. 2025). Nonsteroidal anti-inflammatory drugs (NSAIDs) are alternatively used for the management of non-infectious uveitis because of their relatively favorable safety profile (Guidera et al. 2001), although they are often used as adjunctive therapy because of their relatively weaker anti-inflammatory efficacy compared with glucocorticoids.

Despite the effectiveness of glucocorticoids and NSAIDs in the suppression of immune response, topical instillation via a conventional eye-drop formulation is still a great challenge because of tear flush/drainage and poor trans-corneal permeability (Kaur and Kanwar 2002; Garaszczuk et al. 2018; Lanier et al. 2021). A set of novel ocular drug delivery systems, including mucoadhesive micro/nanoparticles and in situ gelling systems, has been exploited to enhance ocular bioavailability (Li et al. 2018; Teabagy et al. 2023; Sathe et al. 2025; Lei et al. 2025). Among them, ophthalmic gels are the most favorable for the clinical management of various ocular disorders owing to their extended precorneal retention, enhanced drug bioavailability, and ease of operation (Al-Kinani et al. 2018; Wu et al. 2019). Carbomers, as FDA-approved ophthalmic gels, have been widely used for the encapsulation of different therapeutic agents, including NSAIDs (Sullivan et al. 1997; Ceulemans and Ludwig 2002; Kouchak et al. 2019). However, such ophthalmic gel forms an uneven gel layer on the corneal surface after topical instillation, and consequently causes visual blurriness and discomfort (Zhang et al. 2016). Thus, the development of ophthalmic gels capable of achieving effective ophthalmic drug delivery remains an unmet clinical need.

Supramolecular hydrogels derived from amphiphilic small molecules or peptides generally possess unique properties (e.g. self-healing and thixotropy) over polymeric hydrogels, which have gained attention in the field of ocular drug delivery (Chen et al. 2022; Fernandes-Cunha et al. 2022; Pan et al. 2023; Sabbagh et al. 2025). This gel is readily applied to the corneal surface via topical instillation and can be uniformly spread on the corneal surface to resist tear clearance. Additionally, the supramolecular hydrogel underwent ‘gel-sol-gel’ transition on the corneal surface under physiological blinking, which significantly improved the precorneal retention and thus provided better ocular bioavailability (Cheng et al. 2022; Hu et al. 2025). Previous studies have shown that D-form peptide modification of NSAIDs confers its ability to generate a supramolecular hydrogel and boosts its anti-inflammatory capacity (Li et al. 2013; Lin et al. 2023). Inspired by this, we thereafter covalently conjugated an NSAIDs (Pranoprofen; Pra) with a self-assembling D-form tripeptide (i.e. phenylalanine-phenylalanine-aspartic acid; ffd) motif to generate a Pra-peptide conjugate (i.e. Pra-ffd), where the introduction of D-form peptide was expected to enhance the water solubility of Pra and confer the COX-2 selectivity of the conjugate. Specifically, the diphenylalanine (ff) motif was introduced to promote self-assembly through *π*-*π* stacking interactions, whereas the terminal aspartic acid (d) residue was used to finely tune the hydrophilic‒hydrophobic balance of the conjugate, thereby enabling hydrogel formation under physiological conditions. Such a conjugate is supposed to be able to assemble into a single-component supramolecular hydrogel, endowing unique properties (e.g. self-healing, thixotropy), thereby providing the sustained drug retention at the ocular surface relative to free Pra. After topical instillation, the applied supramolecular hydrogel was expected to spread uniformly on the corneal surface, thus minimizing ocular irritation and enhancing drug bioavailability, finally demonstrating its superior therapeutic efficacy in an endotoxin-induced uveitis (EIU) rabbit model.

## 2. Materials and methods

### 2.1. Materials

The 2-chlorotrityl chloride resin and *N*-Fmoc-protected amino acids were purchased from GL Biochem Ltd. (Shanghai, China). Pranoprofen (Pra) was purchased from Aladdin (Shanghai, China). Trifluoroacetic acid (TFA), *N*, *N*, *N*-diisopropylethylamine (DIEA), and triisopropylsilane (TIS) were supplied by J&K Scientific (Shanghai, China). RAW264.7 macrophage and L929 cells were provided by Guangzhou Cellcook Biotech Co., Ltd. (Guangzhou, China). Human Corneal Epithelial Cells (HCEC) were provided by BeNa Culture Collection. Dulbecco’s modified Eagle’s medium (DMEM), DMEM/Nutrient Mixture F-12 (DMEM/F12), and fetal bovine serum (FBS) were purchased from Thermo Fisher Scientific, Inc. (Waltham, MA, USA). The penicillin–streptomycin solution was provided by Biosharp (BL505A, Beijing). 3-[4,5-dimethylthiazol-2-yl]-2,5 diphenyl tetrazolium (MTT) was obtained from Sigma-Aldrich (475989, USA). Lipopolysaccharide (LPS) was purchased from Sigma-Aldrich (St. Louis, MO, USA). Pyrene was purchased from Thermo Fisher Scientific, Inc. (Waltham, MA, USA). Red blood cell lysis buffer (C3702), citrate antigen retrieval solution (P0081), and immunol-staining blocking buffer (P0102) were purchased from Beyotime (Shanghai, China). All other reagents used were of analytical grade.

### 2.2. Synthesis and characterization of Pra-ffd conjugate

As previously reported (Lin et al. 2023), the Pra-ffd conjugate was synthesized using a classical solid-phase synthesis method. The crude product was obtained in a yield of 69.37% and purified by reversed-phase chromatography on a C16 column using a gradient elution of methanol (0.035% trifluoroacetic acid, TFA) and water (0.035% TFA). The purity of the final product was determined to be 94.21%. The identity of the purified conjugate was confirmed by LC–MS (Agilent 1260 Infinity Ⅱ LC with Agilent 6120 MS, Agilent Technologies Inc., USA), FTIR (Tensor Ⅱ, Bruker Optics, Germany), and ^1^H-NMR (Quantum-Iplus, Q. One Instruments Ltd., China).

### 2.3. Fabrication of Pra-ffd hydrogel

The indicated amount of Pra-ffd conjugate was suspended in phosphate-buffered saline (PBS, pH 7.4), and the pH value was adjusted to 7.4 using a sodium carbonate (1M) solution. Subsequently, the resulting mixture was heated to 90 °C for 3 s to generate a clear solution, and the hydrogel spontaneously formed after natural cooling to room temperature.

### 2.4. Characterization of Pra-ffd hydrogel

#### 2.4.1. Transmission electron microscopy (TEM)

The microstructure of the Pra-ffd hydrogel was observed using transmission electron microscopy (TEM, Talos F200S, FEI, USA). A ten-microliter aliquot of hydrogel was pipetted onto a copper grid and then stained with a 5 wt% phosphotungstic acid solution. After drying overnight, the samples were visualized by TEM.

#### 2.4.2. Critical aggregation concentration (CAC)

The CAC value of the Pra-ffd conjugate was measured using a fluorescence spectrometer (FLUOROMAX-4; HORIBA Jobin Yvon, France). Briefly, 40  μL of pyrene solution (6 × 10^−6^ M) in acetone was added to a glass tube, followed by evaporation at room temperature overnight. Next, different concentrations of Pra-ffd aqueous solution were added to the test tube and incubated overnight. The emission spectra of the samples were recorded from 350 to 480 nm using an excitation wavelength of 334 nm. Finally, the CAC value was calculated by plotting the ratio I_em373nm_/I_em384nm_ against their concentrations.

#### 2.4.3. Rheological test

Rheological properties of the Pra-ffd hydrogel were measured using a rheometer (AR2000, TA Instruments, USA). A 300  μL Pra-ffd hydrogel (10  mg/mL) was used for the measurement. A time-sweep test was performed at a constant frequency of 1 Hz and a constant strain of 1%. A frequency sweep test was performed from 0.1 to 100 rad/s at a constant strain of 1%. Strain sweep tests were conducted from 1% to 1000% at a constant frequency of 1 Hz. Amplitude sweep tests were performed at strains of 1% and 100% at a constant frequency of 1  Hz.

#### 2.4.4. *In vitro* stability test

To evaluate the stability of the Pra-ffd conjugate in aqueous solution, 1  mL of Pra-ffd (1  mg/mL) was incubated at 37 °C for the *in vitro* stability study. At predetermined time intervals, 50  μL aliquots were collected, and the Pra-ffd content was analyzed by high-performance liquid chromatography (HPLC, Agilent 1260, Agilent Technologies, USA). The mobile phase consisted of methanol (0.035% trifluoroacetic acid, TFA) and water (0.035% TFA). The eluate was monitored at 220 nm using a diode array detector (DAD).

#### 2.4.5. *In vitro* drug release study

*In vitro* drug release of Pra solution (3.9 mg/mL) and Pra-ffd hydrogel (10 mg/mL) was performed in phosphate-buffered saline (PBS, pH 7.4) at 37 °C. Briefly, 0.2  mL of either Pra solution (3.9  mg/mL) or Pra-ffd hydrogel (10  mg/mL) was sealed into a dialysis bag (molecular weight cut-off: 8000–14,000), followed by incubation in 5  mL PBS containing 0.05% Tween-20 for a period of the *in vitro* drug release study. At specific time intervals, 1 mL of aliquot release medium was withdrawn, and the released drug content was quantified by the HPLC method as aforementioned (Section 2.4.4). At each time interval, 1 mL of the freshly prepared release medium was supplemented.

### 2.5. *In vitro* cytotoxicity

*In vitro* cytotoxicity of the Pra-ffd hydrogel against RAW 264.7 macrophage, L929 cells, and human corneal epithelial cells (HCEC) was assessed using the MTT assay. RAW 264.7 macrophage and L929 cells were cultured in DMEM supplemented with 10% fetal bovine serum (FBS) and 1% penicillin-streptomycin. HCECs were cultured in DMEM/F12 supplemented with 10% fetal bovine serum (FBS) and 1% penicillin‒streptomycin. The cells were seeded in 96-well plates at a density of 1 × 10^4^ cells/well and incubated overnight. After that, culture medium containing different concentrations of Pra-ffd was added for co-incubation. After 24 h of incubation, 20 μL of MTT solution was added to each well and incubated for another 2 h. Formazan crystals were dissolved by the addition of 150 μL dimethyl sulfoxide (DMSO) for 10 min. Finally, the absorbance of each sample was recorded at 490 nm using a microplate reader (Molecular Devices, SpectraMax190, USA). Untreated cells were used as controls. Cell viability was calculated using the following formula: cell viability (%) = (absorbance of sample/absorbance of control) × 100%. All experiments were performed in triplicate (mean ± SD, *n* = 4).

### 2.6. *In vitro* anti-inflammatory assay

Briefly, RAW 264.7 macrophages were seeded in a 24-well plate at a density of 1 × 10^5^ cells/well and incubated overnight. After that, the cells were pretreated with different concentrations of Pra-ffd and Pra (50, 100, and 200  μM) for 24  h, followed by lipopolysaccharide (LPS, 1  μg/mL) challenge for another 24  h. Thereafter, the supernatant from each well was withdrawn, and the levels of interleukin-6 (IL-6) (mean ± SD, *n* = 3), tumor necrosis factor-*α* (TNF-*α*) (mean ± SD, *n* = 3), and prostaglandin E_2_ (PGE_2_) (mean ± SD, *n* = 4) were analyzed using different enzyme-linked immunosorbent assay (ELISA) kits (IL-6, DY406-05, R&D systems; TNF-*α*, DY-410-05, R&D systems; PGE_2_, KGE004B, R&D systems, USA).

### 2.7. Intracellular reactive oxygen species (ROS) measurement

The intracellular ROS levels in RAW 264.7 macrophages were measured using the DCFH-DA assay and flow cytometry, respectively. Briefly, RAW 264.7 macrophages were seeded in a 24-well plate at a density of 1 × 10^5^ cells/well and incubated overnight. The cells were then pretreated with different concentrations of Pra-ffd (50, 100, and 200  μM) for 24 h, followed by challenge with 1  μg/mL LPS for another 24 h. The ROS levels in RAW 264.7 macrophages were visualized by DCFH-DA assay (CA1410, Solarbio, China) using an inverted fluorescence microscope (DMi8, Leica, Germany) and analyzed using a flow cytometer (BD C6 PLUS, USA). Cells that were not manipulated were used as controls. All experiments were performed in triplicate (mean ± SD, *n* = 3).

### 2.8. Animal experiment

#### 2.8.1. Ocular irritation test

New Zealand white rabbits (weighing approximately 2.5 kg) were randomly divided into two groups (*n* = 3 per group) for the ocular irritation test. Fifty microliters of either phosphate-buffered saline (PBS, pH 7.4) or Pra-ffd (10 mg/mL) hydrogel was topically instilled into the conjunctival sac of the eyes three times daily for 7 days. Clinical signs of the eyes, including conjunctival congestion, swelling, secretions, and corneal abnormalities, were observed and graded daily during the entire study by an ophthalmologist using a slip lamp (KangHua®, SLM-4ER, China). Additionally, a fluorescein sodium staining assay was performed to assess the integrity of the corneal epithelium in each group. On the 7^th^ day, the rabbits from each group were sacrificed, and the eyeballs were harvested for histopathological observation using hematoxylin and eosin (H&E) staining.

#### 2.8.2. Precorneal retention test

Eight New Zealand white rabbits (weighing approximately 2.5 kg) were used for the measurement of the precorneal retention of various formulations. To visualize the precorneal retention of the Pra-ffd hydrogel (10 mg/mL) after topical instillation, fluorescein sodium (FS) was physically tagged with the Pra-ffd hydrogel. Fifty microliters of aqueous FS solution or FS-tagged Pra-ffd hydrogel was topically instilled into the conjunctival sac of the eyes. At specific time intervals, green fluorescence on the corneal surface excited by blue light was captured using a slit lamp (KangHua®, SLM-4ER, China) to assess precorneal retention.

#### 2.8.3. *In vivo* pharmacokinetics

Twenty New Zealand white rabbits (weighing approximately 2.5 kg) were employed to assess *in vivo* pharmacokinetics after the topical instillation of Pra solution (3.9  mg/mL) and Pra-ffd hydrogel (10 mg/mL). Briefly, 50 μL of either Pra solution or Pra-ffd hydrogel was topically instilled into the lower conjunctival sac of the rabbits. At specific time points, the rabbits were sacrificed, and the corneas were harvested for homogenization. After the centrifugation at 12,000 rpm/min for 20  min, the supernatant was collected, and the drug content was analyzed by the HPLC method as aforementioned (Section 2.4.4).

#### 2.8.4. Endotoxin-induced uveitis (EIU) rabbit model and medication

According to our previous study (Hu et al. 2022), an EIU rabbit model was established via the intravitreal injection of LPS (100 ng/eye). After LPS intravitreal injection, the rabbits were randomly divided into three groups (*n* = 4 for each group) and treated as follows: (1) topical instillation of 50  μL sterile phosphate-buffered saline (PBS, pH 7.4); (2) topical instillation of 50  μL Pra-ffd hydrogel (10  mg/mL; 15  mM); and (3) topical instillation of 50  μL Pra eye drop (3.9  mg/mL; 15  mM). The rabbits were medicated four times at 3-h intervals. Twenty-four hours later, the clinical signs of EIU in each group were assessed by an experienced ophthalmologist using a slit lamp (KangHua®, SLM-4ER, China) based on established criteria (Table S1) (Zhang et al. 2017). Rabbits without any manipulation were used as the control.

Twenty-four hours later, rabbits from each group were sacrificed, and aqueous humor was harvested for quantification of the number of infiltrated cells and protein level using a hemocytometer and BCA assay (E112, Vazyme Biotech Co., Ltd., China), respectively. Meanwhile, the infiltration of inflammatory cells in the iris-ciliary body from each group was analyzed by flow cytometry (FM4 + , HORIBA, France) using Alexa Fluor 700 CD45 (157616, BioLegend, USA), FITC CD11b (101206, BioLegend, USA), APC F4/80 (123116, Biolegend, USA), and APC/Cyanine7 Gr-1 (108424, Biolegend, USA) antibodies. Additionally, the enucleated eyeball from each group was sectioned for H&E and immunofluorescence staining using CD11b (1:50, 222469, Zenbio, China), ZO-1 (1:200, 617300, Thermo Fisher Scientific Inc., USA), and pan-cadherin (1:50, C1821, Merck, Germany) antibodies. Finally, images of the sections were captured using an LSM 880 confocal microscope (Zeiss, Germany).

### 2.9. Statistical analysis

Data are presented as the mean ± SD. Statistical analyses were performed by one-way analysis of variance (ANOVA) using GraphPad Prism (version 10.1.2, USA). The CAC value was calculated using the Origin software (version 7.5, USA). The maximum drug concentration and area under curve (AUC) was calculated using DAS software2.0 (Shanghai, China). Statistical significance was set at *P* < 0.05.

## 3. Result and discussion

### 3.1. Design, fabrication, and characterization of Pra-ffd hydrogel

Pranoprofen (Pra), a representative of NSAIDs, in the form of an eye drop formulation that has been widely used in ophthalmological practice, but poses several shortcomings, including rapid clearance from the corneal surface and ocular irritation (Liu et al. 2012; Chen et al. 2014). Diphenylalanine peptide (FF), a well-known self-assembling motif, has been extensively utilized as a building block of supramolecular hydrogelator (Levin et al. 2020; Rosa et al. 2022). Bing and co-workers pioneered that the covalent conjugation of D-form Gffy with NSAIDs confers high selectivity for cyclooxygenase-2 (COX-2) (Li et al. 2013). The combination of the D-form ff motif with NSAIDs to construct a drug-peptide supramolecular hydrogel may be a promising strategy to overcome the shortcomings of conventional eye drop formulations. To obtain a pharmacologically relevant hydrogel, we covalently conjugated the D-form tripeptide (i.e. ffd) motif with Pra to generate a Pra-ffd conjugate. In principle, incorporation of the aspartic acid (d) residue significantly enhances the aqueous solubility of Pra and balances the hydrophilic–hydrophobic properties of the conjugate, thereby promoting self-assembly into a supramolecular hydrogel.

**Figure 1.**
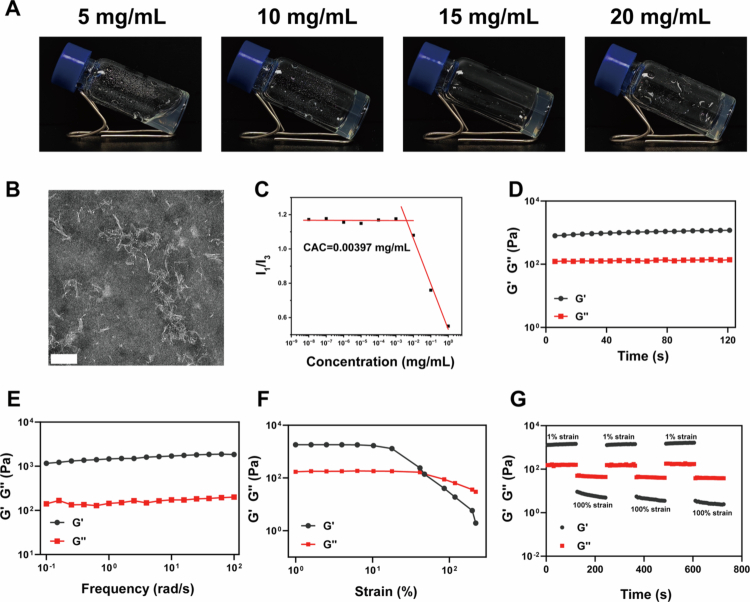
(A) Photography of Pra-ffd aqueous solution at different concentrations; (B) TEM image of Pra-ffd hydrogel (10 mg/mL) (Scan bar: 100 nm); (C) CAC value of Pra-ffd hydrogel; (D) Time sweep of Pra-ffd hydrogel (10 mg/mL); (E) Frequency sweep of Pra-ffd hydrogel (10 mg/mL); (F) Strain sweep of Pra-ffd hydrogel (10 mg/mL); (G) Amplitude sweep of Pra-ffd hydrogel (10 mg/mL).

Based on this concept, the Pra-ffd conjugate was successfully synthesized using a classical solid-phase method. The purity of the conjugate was over 90%, as indicated by LC‒MS analysis, and the identity of the conjugate was confirmed by FTIR and ^1^H-NMR (Figure S1–3). As shown in Figure 1A, it is clearly observed that the Pra-ffd aqueous solution underwent sol–gel transition to afford a transparent hydrogel after a heating–cooling cycle with a minimal gelation concentration of 10 mg/mL. TEM observations (Figure 1B) indicated that the Pra-ffd hydrogel was composed of short nanofibers with an average diameter of 73.67 ± 7.35 nm. The CAC value of the Pra-ffd hydrogel was 0.00397  mg/mL (Figure 1C), indicating its robust self-assembly ability. We then investigated the rheological properties of the Pra-ffd hydrogels. The time sweep test indicated that hydrogelation occurred instantly as its storage modulus (G') increased over the loss modulus (G'') during the entire study period (Figure 1D). The G' value reached a quasi-plateau of approximately 1125 Pa, indicating the formation of a progressively stabilized gel network. The frequency sweep (Figure 1E) indicated the formation of a strong elastic solid network, as the G' value dominated the G'' value in the range 0.1–100 rad/s. The hydrogel collapsed into the sol state when the strain exceeded 50%, implying the stress responsiveness of the hydrogel (Figure 1F). Notably, under low strain (1%), the hydrogel retained its structural integrity, while the G'' value dominated the G' value owing to network disruption at high strain (100%) (Figure 1G). Crucially, the hydrogel recovered immediately upon strain reduction, indicating its thixotropic property. These results collectively indicate that the Pra-ffd hydrogel with favorable viscoelastic characteristics might be a promising candidate for ocular drug delivery.

### 3.2. *In vitro* stability and *in vitro* drug release study

To assess the stability of the Pra-ffd conjugate in aqueous solution, its concentration was monitored over time at 37 °C. As shown in Figure S4, Pra-ffd exhibited no apparent degradation over 28 days, with more than 90% of the conjugate remaining intact. This finding indicated that the Pra-ffd conjugate is relatively stable in aqueous solution and functions as a single-component active molecule. We thereafter compared the *in vitro* drug release of Pra-ffd hydrogel with respect to Pra solution. As shown in Figure S5, the Pra solution exhibited rapid drug release within 2 h, reaching nearly 100% cumulative release, likely due to easy diffusion from the dialysis bag. By contrast, the Pra-ffd hydrogel displayed sustained drug release over a 24 h period, which may be attributed to the viscoelastic properties of the hydrogel.

### 3.3. *In vitro* cytotoxicity and anti-inflammatory ability

**Figure 2.**
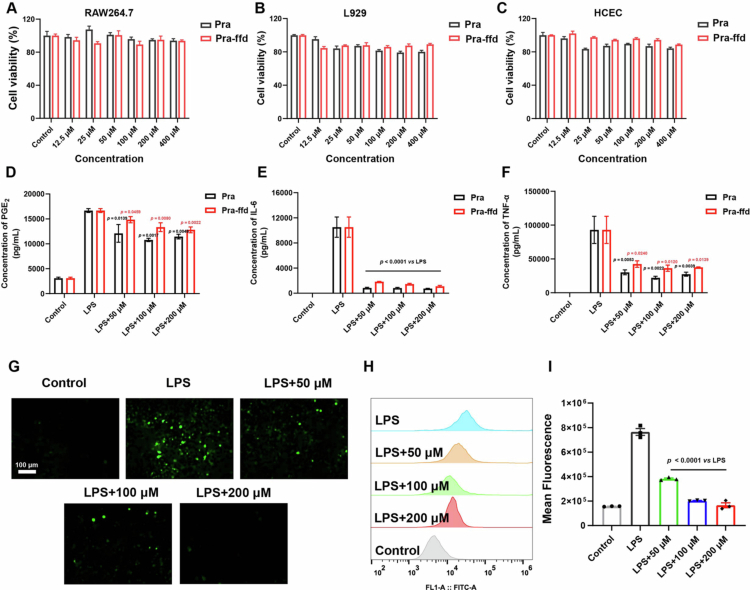
In vitro cytotoxicity of Pra-ffd hydrogel against (A) RAW 264.7 macrophage, (B) L929 cells, and (C) HCEC after 24 h incubation (*n* = 4); (D–F) The impact of Pra-ffd hydrogel on the level of proinflammatory cytokines (PGE_2_, IL‑6, and TNF‑α) in LPS-activated RAW 264.7 macrophage. (PGE_2_: *n* = 4; IL-6: *n* = 3; TNF-α: *n* = 3), *p* *vs* LPS group; (G) Representative fluorescent images of LPS-activated RAW 264.7 macrophage after treatment by different concentrations of Pra-ffd hydrogel; (H, I) Flow cytometry analysis of ROS production in LPS-activated RAW 264.7 macrophage after treatment by different concentrations of Pra-ffd hydrogel (*n* = 3).

In this study, we adopted the MTT assay to assess the *in vitro* cytotoxicity of Pra-ffd on RAW 264.7 macrophage, HCEC, and L929 cells. As shown in Figure 2A–C, both Pra and Pra-ffd caused no apparent cytotoxicity against all tested cells at concentrations below 400  μM. This result suggests that the proposed Pra-ffd has excellent *in vitro* biocompatibility. Similarly, Liu et al. also documented that the NSAID-peptide conjugate exhibited no cytotoxicity against chondrocytes at concentrations below 1  mM (Yang et al. 2025). Thereafter, we used the LPS-activated RAW 264.7 macrophages to evaluate the *in vitro* anti-inflammatory capacity of Pra-ffd. As presented in Figure 2D-F, proinflammatory cytokines, including PGE_2_, IL‑6, and TNF‑α, were significantly elevated after LPS activation. Notably, both Pra and Pra-ffd treatment significantly reduced the levels of proinflammatory cytokines induced by LPS, indicating that peptide modification did not compromise the inherent pharmacological activity of Pra. Singh and co-workers also validated that the NSAIDs-peptide conjugate significantly reduced the expression of proinflammatory cytokines (TNF-*α* and IL-6) in LPS-activated RAW 264.7 macrophages (Halder et al. 2023).

### 3.4. ROS scavenging capability

High ROS levels are generally associated with inflammatory injury, which also plays a vital role in impeding tissue regeneration (Khodr and Khalil 2001; Ogawa et al. 2008). We thereafter investigated the impact of Pra-ffd hydrogel on the level of intracellular ROS in LPS-activated RAW 264.7 macrophage using a DCFH-DA assay. As shown in Figure 2G, LPS-activated RAW 264.7 macrophage exhibited remarkable green fluorescence, indicating an elevation in intracellular ROS levels, as compared to control cells, which displayed invisible green fluorescence. Notably, the Pra-ffd hydrogel dose-dependently quenched the green fluorescence, confirming the outstanding ROS-scavenging capacity of the hydrogel. Quantitative analysis of fluorescence intensity by flow cytometry (Figure 2H, I) also revealed that the intracellular ROS levels were significantly increased after LPS activation, and that Pra-ffd hydrogel treatment remarkably reduced the ROS accumulation induced by LPS in a dose-dependent manner. These findings verified that the proposed Pra-ffd hydrogel is an effective free-radical scavenger.

### 3.5. Ocular irritation and precorneal retention

**Figure 3.**
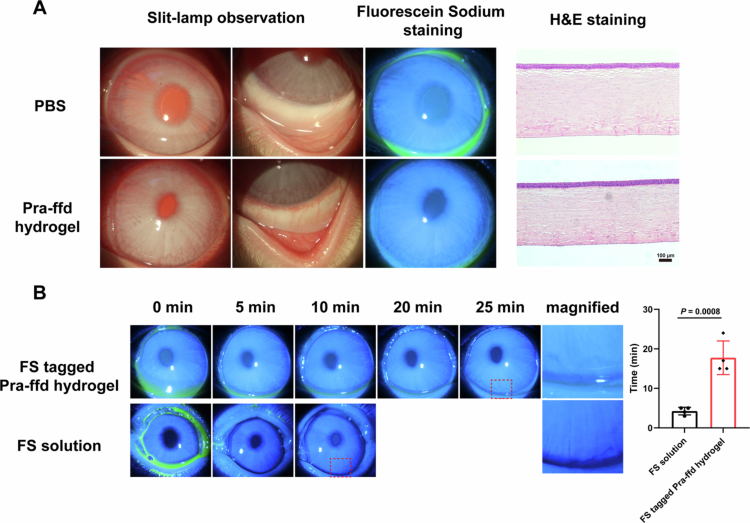
(A) Ocular biocompatibility of rabbit after topical instillation of PBS solution and 10 mg/mL Pra-ffd hydrogel for 7 days; (B) Precorneal retention of fluorescein sodium (FS) solution and FS-tagged Pra-ffd hydrogel (*n* = 4).

Slit-lamp examination in combination with fluorescein sodium staining was used to assess ocular irritation caused by the Pra-ffd hydrogel. As depicted in Figure 3A, after 7 days of treatment, no obvious abnormalities, including anterior chamber inflammation, corneal edema, conjunctival redness, and corneal epithelial injury, were observed in either the PBS or Pra-ffd hydrogel group. Histopathological observations (H&E staining) further verified that the cornea retained its normal architecture in the Pra-ffd hydrogel group. These results indicate that topical instillation of the Pra-ffd hydrogel for 7 days exhibited great ocular tolerance and safety.

Rapid tear flush and turnover result in short precorneal drug retention (generally less than 15 min) on the corneal surface (Lei et al. 2025; Fang et al. 2025). However, an appropriate hydrogel formulation with desirable viscoelastic properties can effectively resist the negative effects of tear flush and consequently extend precorneal retention. As shown in Figure 3B, in the case of fluorescein sodium (FS) solution, the green fluorescence signal in the lower conjunctival sac gradually disappeared approximately 5  min after topical instillation. In contrast, the precorneal retention of the FS-tagged Pra-ffd hydrogel was significantly extended to over 20 min, which was approximately 4-fold greater than that of the solution formulation. The prolonged precorneal retention caused by such a hydrogel might be explained by two aspects: (1) the viscoelastic property of the hydrogel could effectively resist tear flush; (2) the thixotropic character of the hydrogel perfectly fits the stress variation during physiological blinking and thus minimizes ocular irritation and tear production.

### 3.6. *In vivo* pharmacokinetic study

As shown in Figure S6 and Table S2, the maximum drug concentration (C_max_) in the cornea for the Pra solution group was 1.279 ± 0.322 μg/mg, which was significantly lower than that of the Pra-ffd hydrogel group (2.535 ± 0.545 μg/mg). Additionally, the area under the concentration-time curve (AUC_0-6h_) value of the Pra solution group was 0.676 ± 0.171 μg/mg·h, while the Pra-ffd hydrogel group displayed a noticeably higher AUC_0-6h_ (2.441 ± 0.383 μg/mg·h), approximately 3.5-fold higher than that of the Pra solution group, implying the enhanced ocular bioavailability. This might be ascribed to the extended precorneal retention of Pra-ffd hydrogel over that of the Pra solution, thus providing higher bioavailability.

### 3.7. *In vivo* therapeutic efficacy in the EIU rabbit model

**Figure 4.**
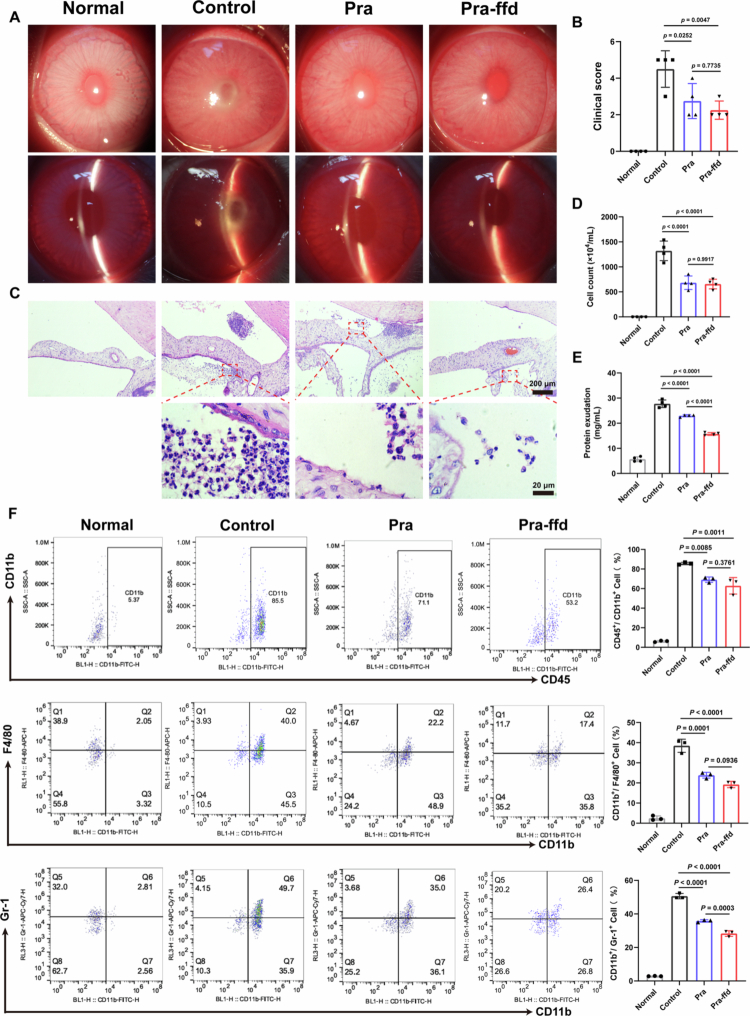
(A) Slit lamp observation of eyes from normal rabbit (Normal), PBS-treated EIU rabbit (Control), Pra eye drop-treated EIU rabbit (Pra), Pra-ffd hydrogel-treated EIU rabbit (Pra-ffd); (B) Clinical score of each group (*n* = 4); (C) H&E sections of anterior chamber from each group; (D) Cell counts in aqueous humor solution from each group (*n* = 4); (E) Protein level in aqueous humor solution from each group (*n* = 4); (F) Flow cytometry analysis of the infiltrated inflammatory cells in iris-ciliary body from each group (*n* = 3).

We thereafter evaluated the therapeutic efficacy of the Pra-ffd hydrogel in an EIU rabbit model. Slit-lamp observation revealed that inflammatory exudate in the anterior chamber was clearly observed in the PBS-treated EIU rabbits (Figure 4A). Conversely, the clinical signs of EIU were significantly alleviated in Pra- and Pra-ffd hydrogel-treated EIU rabbits, corresponding to lower clinical scores with respect to those of PBS-treated EIU rabbits (Figure 4B). H&E staining also revealed numerous inflammatory cell infiltrations in the anterior chamber of the PBS-treated EIU rabbits (Figure 4C). Following Pra and Pra-ffd hydrogel treatment, inflammatory cell infiltration in the anterior chamber was remarkably attenuated, implying robust *in vivo* therapeutic efficacy. Furthermore, the analysis of aqueous humor cell counts and protein levels further demonstrated that Pra and Pra-ffd hydrogel treatment significantly reduced the infiltration of inflammatory cells and the leakage of protein in the anterior chamber compared to PBS-treated EIU rabbit (Figure 4D, E). To identify the infiltration of the cell phenotype in the anterior chamber, flow cytometry analysis was performed. As shown in Figure 4F, there were numerous CD45^+^/CD11b^+^ myeloid cells, CD11b^+^/F4/80^+^ macrophages, and CD11b^+^/Gr-1^+^ neutrophils in the anterior chamber of PBS-treated EIU rabbits, whereas Pra and Pra-ffd hydrogel treatment resulted in a significant reduction of these inflammatory cells in the anterior chamber.

**Figure 5.**
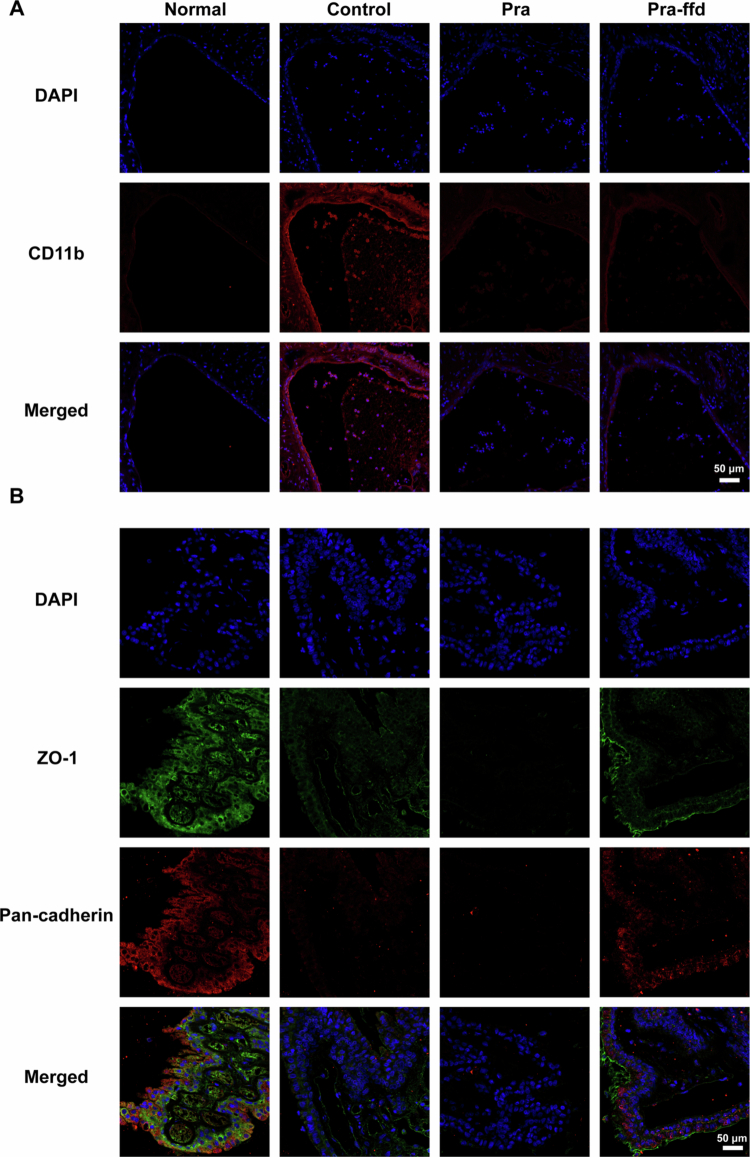
(A) CD11b^+^ cells (red) in the iris-ciliary body of eyes from a normal rabbit (Normal), PBS-treated EIU rabbit (Control), Pra eye drop-treated EIU rabbit (Pra), Pra-ffd hydrogel-treated EIU rabbit (Pra-ffd); (B) ZO-1 (green) and pan-cadherin (red) co-staining in the iris-ciliary body of eyes from each group.

Previous studies have documented that the breakdown of the blood-aqueous humor barrier, resulting in the import of inflammatory cells from the circulation, plays a vital role in the pathogenesis of EIU (Freddo 2013; Alaghband et al. 2019). Immunofluorescence staining indicated the presence of numerous CD11b^+^ myeloid cells in the iris-ciliary body in the PBS-treated EIU rabbits, whereas few CD11b^+^ myeloid cells were observed in the iris-ciliary body in the Pra- and Pra-ffd-treated EIU rabbits (Figure 5A). Thereafter, we assessed the integrity of the blood-aqueous humor barrier in each group using a ZO-1 and Pan-cadherin co-staining assay. As presented in Figure 5B, it is clearly observed that the absence of ZO-1 and Pan-cadherin expression in the iris-ciliary body in the PBS-treated EIU rabbits implies dysfunction of the blood-aqueous humor barrier. Interestingly, Pra-ffd hydrogel treatment rather than Pra treatment protected the integrity of the blood-aqueous humor barrier, as indicated by the expression of ZO-1 and Pan-cadherin in the iris-ciliary body (Figure S7). This result was in accordance with the significant reduction in inflammatory cell infiltration in the anterior chamber by Pra-ffd hydrogel treatment. Overall, these findings demonstrate that the Pra-ffd hydrogel effectively attenuated EIU by protecting the integrity of the blood-aqueous humor barrier.

## 4. Conclusion

In conclusion, we successfully synthesized a drug-peptide conjugate (i.e. Pra-ffd), which could self-assemble into a single-component supramolecular hydrogel with a minimal gelation concentration of 10 mg/mL. The Pra-ffd hydrogel was composed of short nanofibers and exhibited a typical thixotropic property. Moreover, Pra-ffd conjugate hardly caused cytotoxicity against RAW 264.7, HCEC, and L929 cells at concentrations below 400  μM. The Pra-ffd hydrogel dose-dependently attenuated the levels of proinflammatory cytokines (PGE_2_, TNF-*α*, and IL-6) and intracellular ROS accumulation in LPS-activated RAW 264.7 macrophages. Topical instillation of the Pra-ffd hydrogel resulted in great ocular tolerance and precorneal retention over 20 min, thereby enhancing ocular bioavailability with respect to the Pra solution. In an EIU rabbit model, we demonstrated that topical instillation of the Pra-ffd hydrogel effectively mitigated the clinical signs of EIU by reducing the influx of inflammatory cells from the circulation and protecting the integrity of the blood-aqueous humor barrier. Altogether, the proposed Pra-ffd hydrogel with its favorable properties may be a promising strategy for treating various inflammatory ocular disorders. However, several limitations should be noted. In particular, the long-term biocompatibility and *in vivo* safety of the Pra-ffd hydrogel were not fully evaluated in this study and require further investigation. Additionally, the enhanced precorneal retention and sustained drug release of the hydrogel system may reduce dosing frequency and improve patient compliance compared with conventional eye-drop formulations, yet the exact mechanism of its robust anti-inflammatory should be further elucidated.

## Supplementary Material

Supporting information 04 12 Supporting information 04 12.

Author Checklist Full.pdf Author Checklist Full.pdf

## Data Availability

The datasets for this study are available from the corresponding authors upon reasonable request.
