# Supplementary material for: Self-assembled non-steroidal anti-inflammatory drug-peptide hydrogel to effectively mitigate ocular inflammation
Source: Drug Deliv. 2026 May 26;33(1):2677302. doi: 10.1080/10717544.2026.2677302 (PMC13215406; doi:10.1080/10717544.2026.2677302)
Supplement: Supporting information 04 12 [file IDRD_A_2677302_SM7791.docx]

**Supporting information**

**Self-assembled nonsteroidal anti-inflammatory drug-peptide hydrogel to effectively mitigate ocular inflammation**

Yuhan Hu^1^, Yu Li^2^, Yuqin Wu^2^, Yutuo Zhu^2^, Jinrun Chen^2^, Yinghao Ding^1^, Zhimou Yang^*1^, Xingyi Li^*2^

^1^ Key Laboratory of Bioactive Materials, Ministry of Education, College of Life Sciences, State Key Laboratory of Medicinal Chemical Biology, Collaborative Innovation Center of Chemical Science and Engineering, and National Institute of Functional Materials, Nankai University, Tianjin 300071, China

^2^ National Engineering Research Center of Ophthalmology and Optometry, Zhejiang Key Laboratory of Ophthalmic Drug Discovery and Medical Device Research, Eye Hospital, Wenzhou Medical University, 270 Xueyuan Road, Wenzhou 325027, China

^*^Address correspondence to [yangzm@nankai.edu.cn](mailto:yangzm@nankai.edu.cn) (Yang ZM); [lixingyi_1984@mail.eye.ac.cn](mailto:lixingyi_1984@mail.eye.ac.cn) (Li XY)

**Figure captions**

**Fig. S1** LC-MS spectrum of Pra-ffd conjugate

**Fig. S2** ^1^H-NMR spectrum of Pra-ffd conjugate

**Fig. S3** FTIR spectrum of Pra-ffd conjugate

**Fig. S4** LC-MS analysis of Pra-ffd solution (1mg/mL) as function with time at 37 ℃

**Fig. S5** *In vitro* release profiles of Pra solution (3.9 mg/mL) and Pra-ffd hydrogel (10 mg/mL) (n = 4)

**Fig. S6** Drug concentration-time profiles in the cornea after topical instillation of Pra solution (3.9 mg/mL) and Pra-ffd hydrogel (10 mg/mL) (n=4 for each time point)

**Fig. S7** Quantitative analysis of **(A)** ZO-1 and **(B)** pan-cadherin fluorescence intensity in each group (n = 3)

**Table S1**. Scoring system for clinical assessment of uveitis

**Table S2**. Pharmacokinetic parameters in the cornea after topical instillation of Pra solution (3.9 mg/mL) and Pra-ffd hydrogel (10 mg/mL), **p = 0.0074, ***p = 0.0002 *vs* Pra solution, (n = 4)


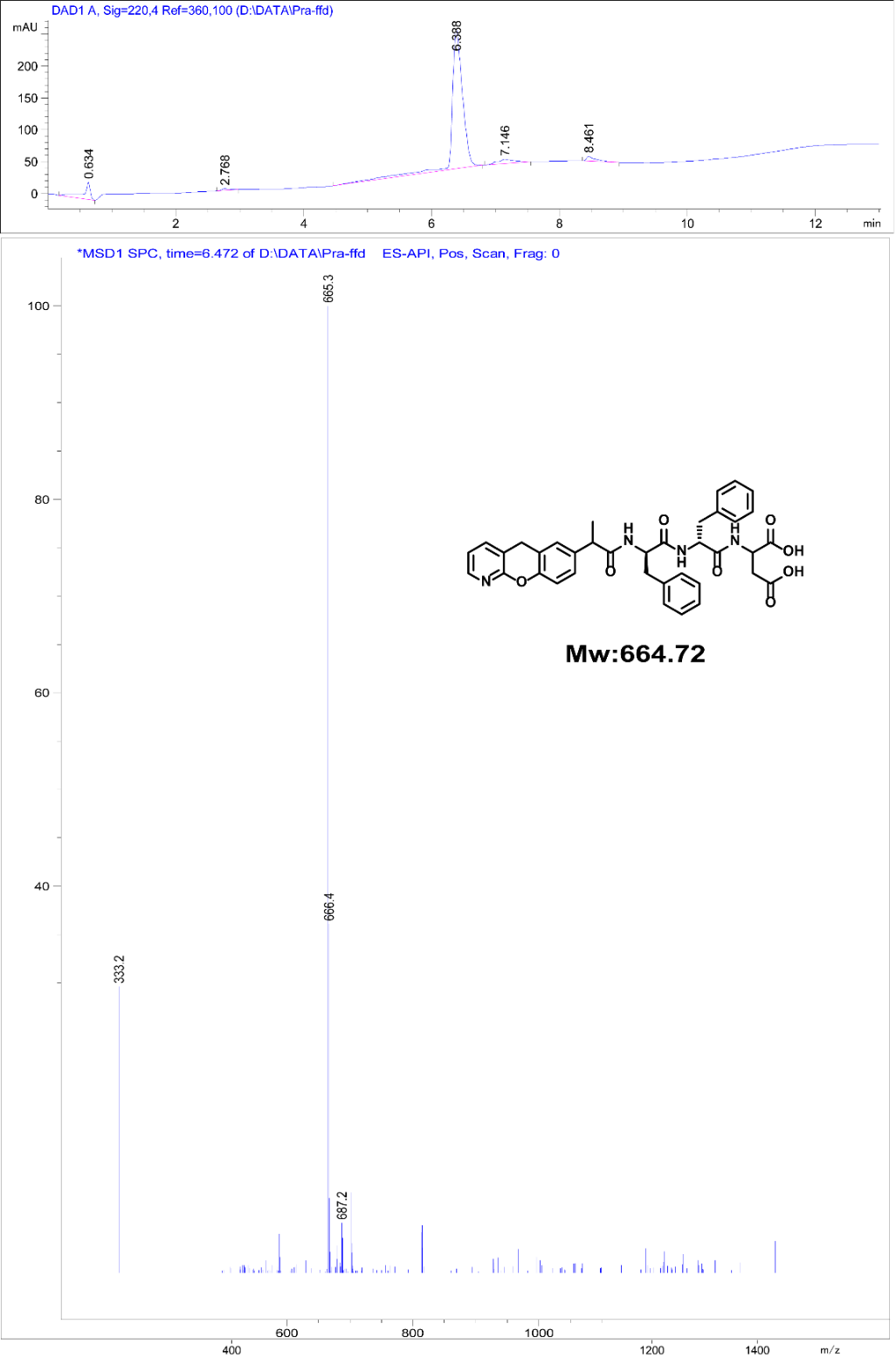


**Fig. S1** LC-MS spectrum of Pra-ffd conjugate


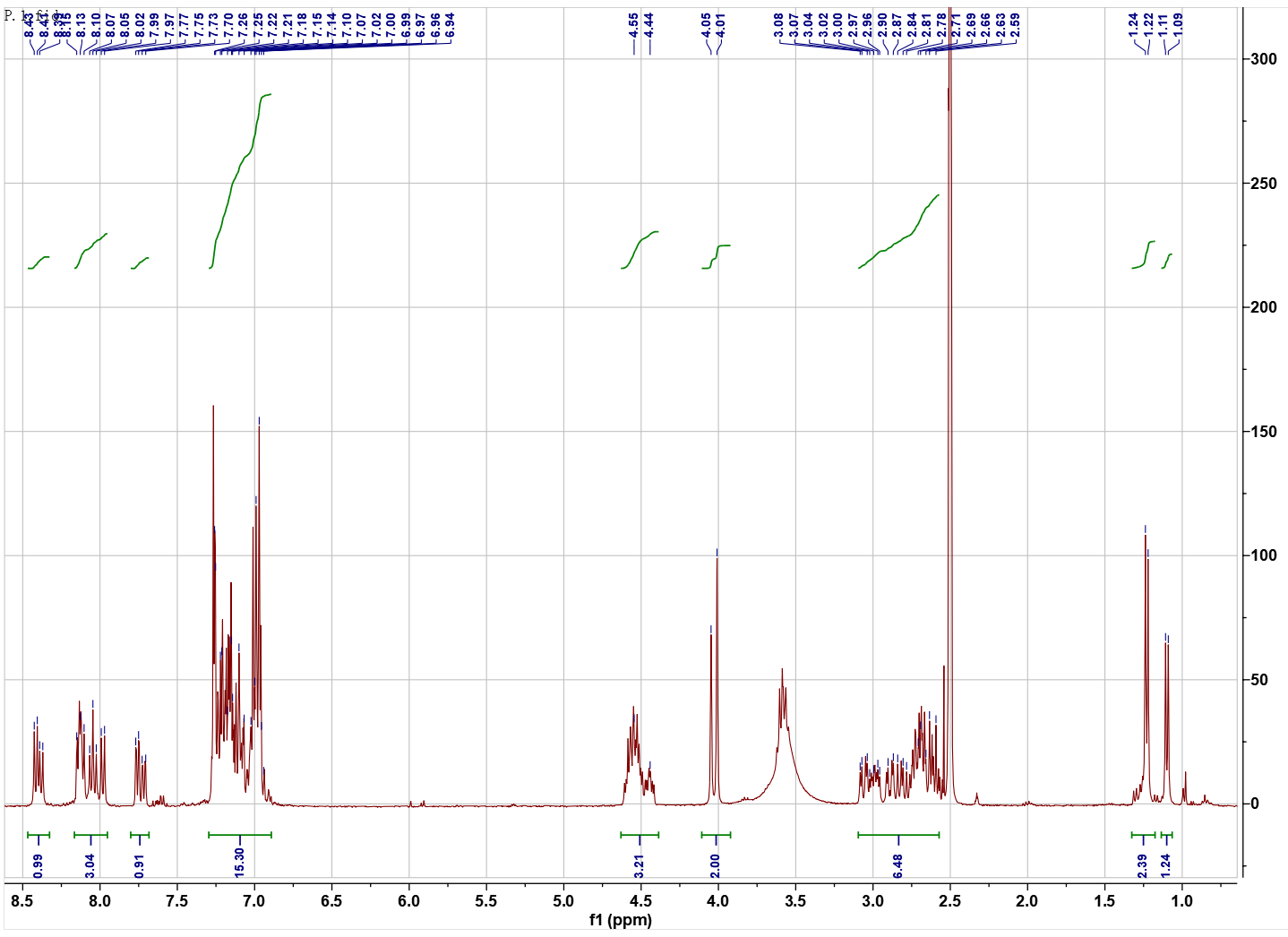


**Fig. S2** ^1^H-NMR spectrum of Pra-ffd conjugate


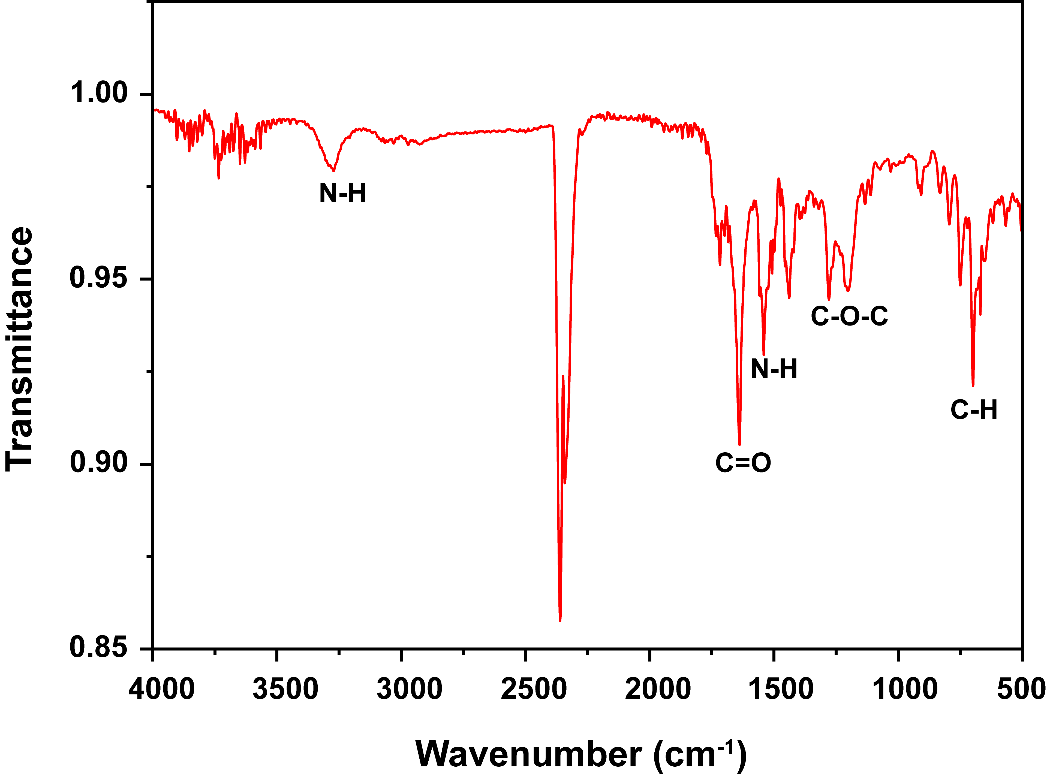


**Fig. S3** FTIR spectrum of Pra-ffd conjugate

**Fig. S4** LC-MS analysis of Pra-ffd solution (1mg/mL) as function with time at 37 ℃

**Fig. S5** *In vitro* release profiles of Pra solution (3.9 mg/mL) and Pra-ffd hydrogel (10 mg/mL) (n = 4)

**Fig. S6** Drug concentration-time profiles in the cornea after topical instillation of Pra solution (3.9 mg/mL) and Pra-ffd hydrogel (10 mg/mL) (n=4 for each time point)


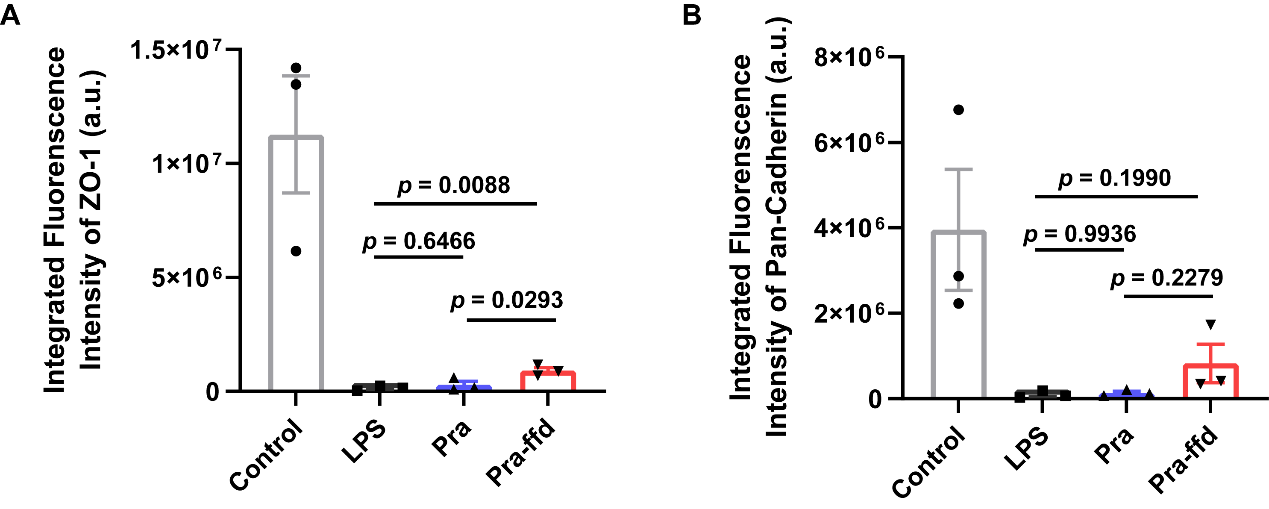


**Fig. S7** Quantitative analysis of **(A)** ZO-1 and **(B)** pan-cadherin fluorescence intensity in each group (n = 3)

**Table S1**. Scoring system for clinical assessment of uveitis

| Clinical signs | Grade of uveitis (score) |
| --- | --- |
| Iris hyperemia |  |
| Absent | 0 |
| Mild | 1 |
| Moderate | 2 |
| Severe | 3 |
| Pupil |  |
| Normal | 0 |
| Miosis | 1 |
| Exudate in anterior chamber |  |
| Absent | 0 |
| Small | 1 |
| Large | 2 |
| Hypopyon |  |
| Absent | 0 |
| Present | 1 |
| Maximum possible score | 7 |

**Table S2**. Pharmacokinetic parameters in the cornea after topical instillation of Pra solution (3.9 mg/mL) and Pra-ffd hydrogel (10 mg/mL), **p = 0.0074, ***p = 0.0002 *vs* Pra solution, (n = 4)

|  | C_max_ (μg/mg) | AUC_0-6 h_ (μg/mg·h) |
| --- | --- | --- |
| Pra solution | 1.279 ± 0.322 | 0.676 ± 0.171 |
| Pra-ffd hydrogel | 2.535 ± 0.545** | 2.441 ± 0.383*** |
